# The Twofold Role of Osteogenic Small Molecules in Parkinson's Disease Therapeutics: Crosstalk of Osteogenesis and Neurogenesis

**DOI:** 10.1155/2022/3813541

**Published:** 2022-12-12

**Authors:** Shima Tavakol

**Affiliations:** Cellular and Molecular Research Center, Iran University of Medical Sciences, Tehran, Iran

## Abstract

Deemed one of the most problematic neurodegenerative diseases in the elderly population, Parkinson's disease remains incurable to date. Ongoing diagnostic studies, however, have revealed that a large number of small molecule drugs that trigger the BMP2-Smad signaling pathway with an osteogenic nature may be effective in Parkinson's disease treatment. Although BMP2 and Smad1, 3, and 5 biomolecules promote neurite outgrowth and neuroprotection in dopaminergic cells as well, small molecules are quicker at crossing the BBB and reaching the damaged dopaminergic neurons located in the substantia nigra due to a molecular weight less than 500 Da. It is worth noting that osteogenic small molecules that inhibit Smurf1 phosphorylation do not offer therapeutic opportunities for Parkinson's disease; whereas, osteogenic small molecules that trigger Smad1, 3, and 5 phosphorylation may have strong therapeutic implications in Parkinson's disease by increasing the survival rate of dopaminergic cells and neuritogenesis. Notably, from a different perspective, it might be said that osteogenic small molecules can possibly put forth therapeutic options for Parkinson's disease by improving neuritogenesis and cell survival.

## 1. Introduction

One of the most significant signaling pathways in osteogenesis is the BMP signaling pathway, which triggers a phosphorylation cascade of R-Smads to induce overexpression of the genes involved in osteogenesis. It is also worth noting that ubiquitination plays a critical role in the inhibition and activation of signal transduction by Smads. In brief, Smad ubiquitination regulatory factor 1 (Smurf1) undergoes phosphorylation at S148 by AMP-activated protein kinase (AMPK). Smurf1 is an HECT-type ubiquitin ligase that belongs to the Nedd4 subfamily [1]. It has a calcium-binding C2 domain along with 2-4 WW (tryptophan-tryptophan) domains and is bound to the target proteins through the latter. The phosphorylated form of Smurf1 ubiquitinates Smad1 and Smad5, resulting in Smad degradation through the 26S proteasome. Moreover, it is able to directly ubiquitinate Runx2 as an early osteogenic biomolecule to inhibit osteogenesis. Additionally, Smurf1 is bound to the PY motif of Smad6 in the linker region, a negative osteogenic regulator, and translocates it from the resting state in the nucleus to the active state in the cytoplasm, hindering osteogenesis [2].

Smads are categorized into 3 types called R-Smad, co-Smad, and I-Smad based on their structural and functional differences. Smad1, 3, 5, and 8 are R-Smads. Samd4 is the co-Smad. I-Smad includes Smad6, which inhibits BMP and Smad signaling, and Smad7, which inhibits BMP, TGF-*β*, and activin signaling. Moreover, Smads have two distinct domains called MH1 and MH2 with a linker region between them. MH1 prevents the biological activity of MH2. Although R-Smads and Smad4 have similar structures, Smad4 is not phosphorylated by type I receptor kinase. Smad1, 5, and 8 are substrates of BMPs, while Smad2 and 3 are substrates of TGF-*β*, nodal, and activin. Smad1, 3, 5, and 8 are phosphorylated by activated type I receptor kinases. The phosphorylation site of Smad3 is an L3 loop in the MH2 domain (Asn240, Gln241, Arg287, and His288) [3], which is located in the C-terminal SXS motif. It is phosphorylated by the L45 loop of the GS domain in ligand-activated TGF-*β* receptor kinases [4]. Smad anchor for receptor activation (SARA), located in the cell membrane, binds to monomeric Smad3 (Lys332, Lys377, and His288 in the L3 loop) through its Smad-binding domain (SBD) and Glu607 (amino acids located downstream of the FYVE domain (double zinc finger domain)). It then phosphorylates Smad3 by receptor kinases that are located in the SARA carboxy-terminal domain. Furthermore, SARA recruits R-Smads in the cell membrane where type I receptors are present. This phenomenon leads to the dissociation of Smad3 from SARA and its complex formation with Smad4. Since I-Smads and co-Smad do not have an SXS motif on the MH2 domain extreme, they are not phosphorylated by activated type I receptors. Additionally, Smad4 has a special insertion element that prevents its interaction with type I receptors.

It is worth noting that the MH1 domain located in the N-terminus of Smads has sequence-specific DNA binding activity and binds to palindromic DNA sequences (Smad-binding element (SBE); 5′-GTCTAGAC-3′) through its *β*-hairpin structure [5–7]. Since I-Smads have short sequences in the MH1 domain, they cannot bind to DNA.

Short-term BMP2 upregulation induces irreversible osteogenesis by Smad1, 5, and 8 phosphorylation [8]. With an impact similar to that of BMP2, TGF-*β*1 prompts Smad3 phosphorylation, which is essential for bone formation, by decreasing osteoblast apoptosis with the aim of mineralization and increasing ALP activity [9]. Therefore, it might be said that any factor that inhibits Smurf1 phosphorylation and activation or triggers Smad1, 3, and 5 phosphorylation will result in osteogenesis either directly or indirectly. The direct impact will be through Runx2; whereas, the indirect path, among others, will be the translocation of the concomitant phosphorylated Smad1, 3, and 5 into the nucleus through interaction of the MH2 domain of R-Smads with the basic amino-acid-rich region in the MH2 domain of Smad4 [10] for the osteogenic gene transcription of Runx2 and osteocalcin [11]. In addition, Smad1 and 5 have a PY motif in the linker region that binds the WW1 motif of Smurf1 to four (binding) sites for phosphorylation by glycogen synthase kinase-3 (GSK3) and cyclin-dependent kinase-8 (CDK8). After translocation into the nucleus and binding to the genome, Smad1 and 5 are again phosphorylated by GSK3, which results in their capture by Smurf1 through the PY motif. This cascade will repeat itself to maintain bone homeostasis [12]. Apart from Smad phosphorylation, Smad acetylation by CBP and p300 at Lys-378 in Smad3 is effective in R-Smad activation [5].

Recently, there has been a growing interest among scientists to use small molecules (with low molecular weight, less than 1000 Da) [13–17] instead of biomolecules [18], biomaterials [19–21], growth factors, and recombinant biomolecules in therapeutic studies. Small molecules are synthetic organic compounds that modulate signaling pathways by mimicking special biomolecules and growth factors. Among their features, which make them appealing in pharmaceutical and regenerative medicine, are the possibility of large-scale production, in vivo chemical stability, low cost, fewer side effects in the process of dose decrement, oral administration, and a nonimmunogenic and pyrogenic nature [22, 23].

When Smad1, 3, and 5 phosphorylation and nuclear translocation are triggered using osteogenic small molecules, osteoblast cell metabolic activity, which is a marker of cell viability, increases. Subsequently, the genes involved in osteogenesis, such as Runx2, alkaline phosphatase (ALP), type I collagen, osteonectin, and osteocalcine, are overexpressed. The overexpression of osteogenic genes leads to the upregulation of their proteins and calcium deposition by differentiated osteoblasts, an increase in ALP enzyme concentration and activity, and an increase in collagen content, all of which eventually result in osteogenesis and bone repair [24].

It is worth noting that the FDA has approved a number of small molecule drugs for a group of medical ailments, such as bone fractures. Doxycycline, simvastatin, rapamycin, alendronate, retinoic acid, FK506, dexamethasone, and vitamin D are included in the FDA-approved list of drugs. The main focus of the present study was on small molecules that mimic Smurf1 inhibitors and Smads as the two critical arms of the BMP2 signaling pathway in osteogenesis.

## 2. Small Molecules in Osteogenesis

### 2.1. Smurf1 in Osteogenesis

Recent discoveries have shed light on a number of small molecules; the signaling pathways of which have yet to be completely identified are being reported as osteogenic small molecules with BMP2 upregulations, such as 2-((1-(Benzyl(2-hydroxy-2- phenylethyl)amino)-1-oxo-3-phenylpropan-2-yl)carbamoyl) benzoic acid. The aforementioned small molecule has proven to be effective in inducing the overexpression of Runx2, type I collagen, BMP2, and osteocalcin in vitro, osteoblast recruitment and formation, mineralized tissue formation, and proteasome activity inhibition in bone defects in a rat model [25]. Mund et al. [26] disclosed a bicyclic peptide small molecule that selectively binds to Smurf2 but not to Smurf1 and changed its conformation to inhibit ubiquitination. Another small molecule involved with Smurf1 is phenamil, which, through tribbles homolog 3 (Trb3) overexpression in cells, particularly degrades Smurf1 protein and suppresses Smurf1 gene expression while stabilizing Smads and inducing their overexpression in cells. Additionally, it induces overexpression of Runx2, ALP, type I collagen, osteocalcine, and osterix genes while inducing calcium deposition in osteodifferentiated MSCs [27]. Not only phenamil induces bone repair in vivo but also by Trb3 upregulation; it inhibits inflammation and cyst formation in defect site [28]. Furthermore, Cao et al. [12] reported the two chemical formulae C_22_H_20_C_l_F_3_N_4_O_3_S and C_25_H_26_FN_3_O_4,_ which are located in the WW1 pocket of Smurf1 and inhibit Smad1 and 5 ubiquitination to induce BMP signaling and ALP activity in osteoblast-like cells. Additionally, the two small molecules isoliquiritigenin and 4′-hydroxychalcone increase Smad1 and 5 phosphorylation by decreasing Smad ubiquitination to promote osteogenesis [29].

### 2.2. Smads' Small Molecules in Osteogenesis

Apart from affecting Smurf1 directly, a number of small molecules influence Smads through other hot points, such as alendronate, which upregulates BMPR2, BMP, and Smad3 as the genes involved in BMP signaling to induce osteogenesis significantly [30]. As another effective small molecule, FK506 [24] improves bone regeneration through interaction with BMPR2 and then with BMPR1 and Smad1 and 5 phosphorylation. It also increases parathyroid hormone in the serum and pyridinoline in the urine of rats. Another small molecule to be taken into account is sirolimus (rapamycin), which induces osteogenesis by increasing pSamd3 and Smad4 upregulation [23]. The final small molecule to be mentioned here is PGE-5516909, which prompts Smad1C to increase ALP activity and bone regeneration. Overall, the prospect of bone regeneration by small molecules seems to be focused on the role that Smad activators and Smurf1 inhibitors play in the process, which is partly similar to the course of studies in Parkinson's disease therapeutics.

## 3. Osteogenic Small Molecules in Parkinson's Disease Treatment

Considering the ongoing growth of the elderly population worldwide, Parkinson's disease is increasing as the second most common neurodegenerative disease. In Parkinson's disease, the dopaminergic neurons located in the substantia nigra pars compacta undergo apoptosis, resulting in a decrease in the level of dopamine in the brain [31]. Two of the earliest occurrences in Parkinson's disease pathology are believed to be dopaminergic axonal retraction and degeneration with concomitant innervation loss, while complete dopaminergic somal death is postponed. It is worth mentioning that axonal regeneration is as important as soma survival regardless of the different mechanisms that are involved in their etiology [32].

It is essential to take into account that symptoms such as depression, dystonia, anxiety, tremor, and dyskinesia in Parkinson's disease patients are related to the deficiency of neurotrophic factors and are not the direct outcome of dopamine depletion. Unfortunately, despite significant progress in the global cognizance of Parkinson's disease pathology and advances in pharmaceutical technologies, there is no definitive cure for the disease. Clinical trials involving GDNF as a critical neurotrophic factor in dopaminergic neuron survival have failed due to the downregulation of Ret, a GDNF coreceptor, induced by *α*-synuclein in Parkinson's disease studies [33]. Therefore, finding small molecules that are Ret-independent and, as a result, helpful in stimulating neurotrophic factors, improving dopaminergic neuron survival, dopamine synthesis, and diminishing neuronal apoptosis has been the focus of many intensive studies.

Other than the benefits mentioned so far, small molecules that can mimic the function of BMP proteins are also sought as valuable agents since BMP proteins are rapidly metabolized in the brain [34]. Another point is that in order for the drugs to penetrate the brain, they must cross the blood-brain barrier (BBB). The BBB is disrupted and more permeable to drugs and toxins in Parkinson's disease patients due to downregulated tight junction proteins and low functionality of P-gp [35]. P-gp dysfunctionality is said to result in an increase in bioavailability of the drugs in the brain up to 150-fold [36].

As mentioned earlier, small molecules are organic compounds with molecular weights of less than 1000 Da [13]. These findings indicate that compounds with a molecular weight less than 500 Da freely diffuse through the intact BBB; however, the possibility of diffusion decreases 100-fold when the molecular weight increases from 200 to 450 Da. In addition, molecular volume, lipophilicity (log P: 1.5–2.5), and polar surface area (60-90 Å) influence the permeation of small molecules [35]. Apart from polar surface area, small molecules with significant positive or negative electrostatic charge are not favored for passive diffusion across the BBB. Moreover, the hydrogen bond donors and acceptors of the drug with water must be less than 5 and 10, respectively, and the small molecules are preferred to have 5 or fewer rotatable bonds to cross the BBB [35]. Taking these properties into account while screening small molecules helps scientists in Parkinson's disease therapeutic studies.

Considering the growing number of osteogenic small molecules involved in Smad phosphorylation that have been reported in various studies, those engaged in neuritogenesis, dopamine synthesis, and dopaminergic cell survival, might put forth the next generation of therapeutic opportunities for Parkinson's disease. Therefore, the remaining portion of this paper will focus on the influence of Smurf1 and Smad small molecules on dopaminergic neurons with the aim of exploring prospective therapeutic opportunities.

To elaborate on the BMP processes mentioned above, BMP2 and 7 systematically activate BMP receptors such as BMPR2 and BMPR1b, which in turn, phosphorylate Smads. BMPR2 and BMPR1b are expressed in dopaminergic neurons and increase their cell survival [35]. Meanwhile, a rise in BMPR2 itself results in Smad1 and 5 phosphorylation along with neuritogenesis in dopaminergic neurons [37]. However, pretreatment of embryonic dopaminergic cells with BMP2 and BMP7 increases the viability of dopaminergic cells in the lesion site of the striatum [35].

### 3.1. Role of Smurf1 Small Molecules in Parkinson's Disease Treatment

**Figure 1:**
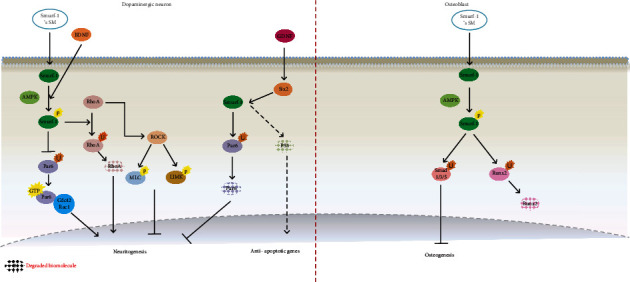
Schematic representation of crosstalk between dopaminergic neurons and osteoblasts induced by small molecules involved in the Smurf1 signaling pathway is shown. (a) This picture highlights the effect of Smurf1 mimicking small molecules in neurogenesis. Induction of p-Smurf1 led to RhoA ubiquitination and resulted in less ubiquitinated Par6 in a competitive manner. This cascade along with cdc42 and Rac1-induced neurite outgrowth. However, RhoA, through the activation of Rho-associated protein kinases (ROCKs), phosphorylates myosin light chain (MLC) and LIMK, both of which separately phosphorylate cofilin. p-cofilin depolymerized actin and inhibited neuriotogenesis. In addition, GDNF, through an increase in Six2, degrades the P53 protein and induces Bcl2 overexpression, which leads to cell viability improvement. (b) This picture highlights the effect of Smurf1 mimicking small molecules in the inhibition of osteogenesis. Induction of Smurf1 and subsequent Smurf1 phosphorylation led to Runx2 degradation and osteogenesis inhibition.

Due to the critical role that neuritogenesis plays in Parkinson's disease therapeutic procedures, it needs to be analyzed in depth. Smurf1 phosphorylation, which is induced by BDNF, cAMP, and protein kinase, partially defines neuritogenesis [38, 39]. Following Smurf1 phosphorylation, pSmurf1 competitively ubiquitinates RhoA instead of Par6, and the degraded RhoA results in axon growth derived from Par6 stabilization. In addition, nonphosphorylated Smurf1 competitively ubiquitinates Par6 and induces Par6 degradation, which causes actin growth inhibition by actin cytoskeleton modulation in an affinity-binding manner [39, 40]. Furthermore, Smurf1 phosphorylation at T306 in neurons induces substrate preference characteristics resulting in Par6 translocation to the tip of the axon and, subsequently, neuritis growth due to Cdc42 and Rac1 [39, 40]. Meanwhile, phosphorylated Par6 ubiquitinates RhoA through the TG*β* receptor (T*β*R2) and induces neuritogenesis. Apart from its role in neuritogenesis, Smurf1 inhibits apoptosis of damaged dopaminergic neurons through p53 degradation when it is upregulated by mediation of the transcription factor Six2 [41]. The role of Smurf1 in neuritogenesis is in contrast with its role in osteogenesis, while Smurf1 phosphorylation favors neuritogenesis, its phosphorylation inhibits osteogenesis by Samd1 and 5 ubiquitination [42]. Based on these findings, unlike bone regeneration, small molecules that induce Smurf1 phosphorylation are valuable in Parkinson's disease therapeutics (Figure 1).

### 3.2. Role of Smad Small Molecules in Parkinson's Disease Treatment

In a study, Alexanian et al. [43] reported that small molecule inhibitors of Smad1, 5, and 8 generate neuron-like cells derived from human mesenchymal stem cells, which significantly upregulate Nurr1 and TH proteins as the two markers of dopaminergic cells. Another study conducted by Ladewig et al. [44] revealed that TGF-*β*1-Smad inhibition eventually converts human postnatal fibroblasts into TH-positive neuron-like cells through the mesenchymal-to-epithelial transition (MET) phenomenon. These studies have disclosed the fact that inhibition of Smad1 and Smad5 favors dopaminergic neuron transdifferentiation.

Recent studies have shown that Smad3 deficiency leads to the catabolism of dopaminergic neurons, a decrease in trophic support, an increase in oxidative stress, and *α*-synuclein aggregation, which together intensify Parkinson's disease symptoms [45]. Therefore, as is the case in osteogenesis, Smad3 might also be considered a hot point in Parkinson's disease treatment through apoptosis inhibition of dopaminergic and osteoblast cells with small molecules such as pitavastatin (MW: 421.468 g/mol; LogP: 2.92), mevastatin (MW: 390.52 g/mol; LogP: 3.95), and simvastatin (MW: 418.574 g/mol; LogP: 4.46) from the statin family [9].

**Figure 2:**
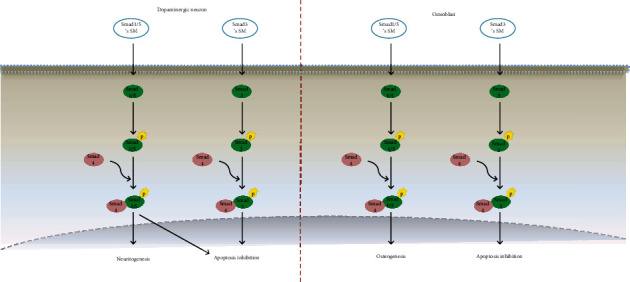
A schematic representation of the crosstalk between dopaminergic neurons and osteoblasts induced by small molecules involved in the Smad signaling pathway is shown. (a) This picture highlights the effect of Smad1/3/5 mimicking small molecules in neurogenesis and apoptosis inhibition. Induction of Smad 1/5 triggers a cascade that leads to the interaction of Smad4 as a co-Smad with p-Smad 1/5 and translocation of the complex into the nucleus and neurites. Small molecules mimicking Smad 3 resulted in the formation of the Smad4-p-Smad3 complex and its translocation into the nucleus and inhibition of neural apoptosis. (b) This picture highlights the effect of Smad1/3/5 mimicking small molecules in osteogenesis and apoptosis inhibition. The previously mentioned cascade in dopaminergic neurons occurs in osteoblasts. Smad1/5 mimicking small molecules triggers the cascade that leads to osteogenesis, while Smad3 mimicking small molecules triggers the cascade that leads to apoptosis inhibition in osteoblasts.

Studies have shown that phosphorylation of Smad1, 5, and 8 increases the survival rate of damaged retinal ganglion cells [46] and a number of dopaminergic neurons [47], along with the rate of neurite outgrowth [48]. Additionally, pSmad1 promotes neuritogenesis through transcriptional regulation of Erk1/2 [49]. Meanwhile, through Smad1, BMP2 induces the differentiation of enteric neurons to TH-positive neurons, which are known as dopaminergic neurons [50]. Furthermore, CTPB (N-(4-chloro-3-trifluoromethyl-phenyl)-2-ethoxy-6-pentadecyl-benzamide), (MW: 554.135 g/mol), as a small molecule, activates BMP-Smad signaling through interaction with Smad1 and Smad4. It increases neuritogenesis and survival of dopaminergic neurons the same way a neurotrophic factor does [51]. Therefore, even though Smad1 and 5 inhibitors induce transdifferentiated dopaminergic neurons, phosphorylated Smad1 and 5 induce dopaminergic neuron survival and neurite outgrowth (Figure 2).

There are a number of studies on the effect of the osteogenic small molecule FK506 (MW: 804.018 g/mol, LogP: 2.74) in animal models of Parkinson's disease in which the neuroprotective potential, the increase in TH-positive cells, and the increase in motor strength of FK506 have been attributed to the phosphatase activity modulation of calcineurin [52]. However, none of these studies has reported any findings on the role of Smad signaling pathways, which has been proposed by the author of this article and calls for a thorough study.  Table 1 shows crosstalk of osteogenic small molecules involve in Smad1/3/5, BMP2, and Smurf1 signaling pathway.

## 4. Targeted Drug Delivery in Parkinson's Disease

Small molecules which have a low molecular weight could easily cross the BBB. However, as mentioned earlier, other parameters such as LogP and polar surface area are important. To overcome these drawbacks, targeted drug delivery using nanocarriers is promising. In other words, passive targeting is based on the size and physical properties of a drug that facilitates the transportation of drug into a target tissue based on pore vascularity and etc. while active targeting is based on chemical decoration of a nanocarriers to the target cell. Drug nanocarriers enhances bioavailability, solubility, chemical stability, biodegradation, circulation half-life, ease of surface targeting, and permeation of drug while decreases effective dose resulting in less side effects in other tissue and organs [53, 54]. For example, lipid nanoparticles containing cholesterol improve the stability of drug and cell-membrane fusion [55]. Solid lipid nanoparticles (SLNs) and nanostructured lipid carriers (NLCs) have some advantage compared to the liposomes such as higher loading capacity, bioavailability, and control on drug release while SLN during the time crystallize in storage site and expulse the drug. Therefore, NLC is preferred to SLN [56]. Zhao et al. synthesized a lipid nanoparticle (167 nm, EE 86.7%) containing bFGF as a targeting agent for the brain, delivering drugs to the striatum of on by serum proteins. Results were promising when compared to the naked-bFGF nanocarriers [57]. PLGA-albumin nanoparticles containing dopamine showing enhanced permeability to the brain through the interaction of albumin with special cell surface receptors in Parkinson's disease model in mice [58]. Another strategy for substantia nigra and striatum targeting is conjugation of the lipid nanoparticles to the RVG29 peptide which is a rabies virus glycoprotein [59]. Other receptors that can be targeted to deliver drugs through BBB would be insulin, lactoferrin, transferrin, scavenger, diphtheria toxin, lipoprotein, folate, and choline receptors [60]. Overall, nanocarriers' conjugation with ligands that target special cell surface receptors on neurons and BBB is promising to deliver small molecules to substantia nigra and striatum.

## 5. Outline

In conclusion, and as it has been reiterated throughout the present study, osteogenic small molecules are considered prime candidates in Parkinson's disease therapeutic studies considering their low molecular weight in LogP screening, polar surface area, electrostatic charge, and hydrogen bond donor and acceptor status, which enables them to cross the BBB freely [35]. It is worth noting that osteogenic small molecules that inhibit Smurf1 phosphorylation do not offer therapeutic opportunities for Parkinson's disease; whereas, osteogenic small molecules that trigger Smad1, 3, and 5 phosphorylation may have strong therapeutic implications in Parkinson's disease by increasing the survival rate of dopaminergic cells and neuritogenesis.

**Figure 3:**
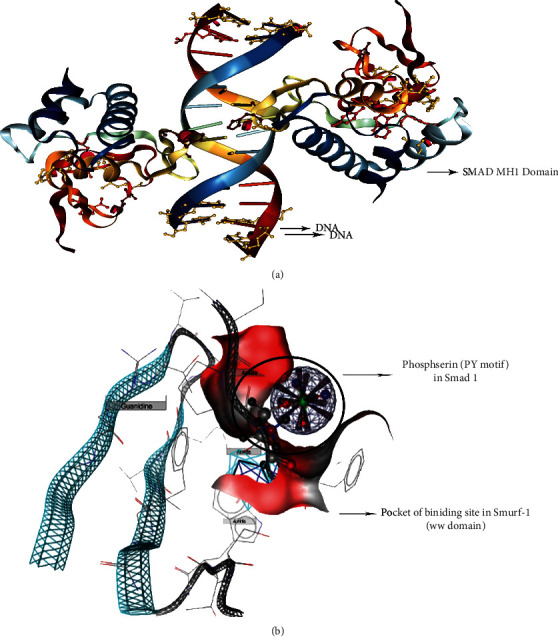
(a) Smad MH2 domain bound to DNA (PDB: 1MHD). (b) PY motif in the linker region of Smad1 bound to the WW domain of Smurf1. The small molecule that occupies this pocket of the binding site inhibits Smad1 ubiquitination (PDB: 2LAZ).

Another strategy is to design or screen a small molecule that, in addition to having the abovementioned properties, can be located in the WW1 pocket of Smurf1 to inhibit Smads ubiquitination or small molecules that phosphorylate Smurf1 at T306 [39, 40] and T331 [61] to stabilize and translocate par6. In such cases, it can also decrease its binding affinity with ligands containing PY motifs (Smads) with the aim of neurogenesis and inhibition of Smad ubiquitination. Taking into account the fact that amino acid sequences have been highly conserved in the Smad family and their mutation results in aberrant normal pathways [4], targeting specific motifs (SXS in the MH2 domain) in Smad1, 3, and 5 to be phosphorylated using osteogenic small molecules might be the key point in Parkinson's disease therapeutic studies. Moreover, it is valuable to screen osteogenic small molecules that induce phosphorylation of the linker region at S206 and S214 in Smad1 and T179 in Smad3 [4] to activate Smad signaling pathways and eventually promote neuritogenesis. Another important thing is to monitor the small molecules that occupy the PS-TP and/or PY motifs in the linker region of R-Smads to induce Smad accumulation in the nucleus and ubiquitination inhibition (Figure 3) through prevention of MAPK phosphorylation and Smurf ubiquitination, respectively [7].

**Figure 4:**
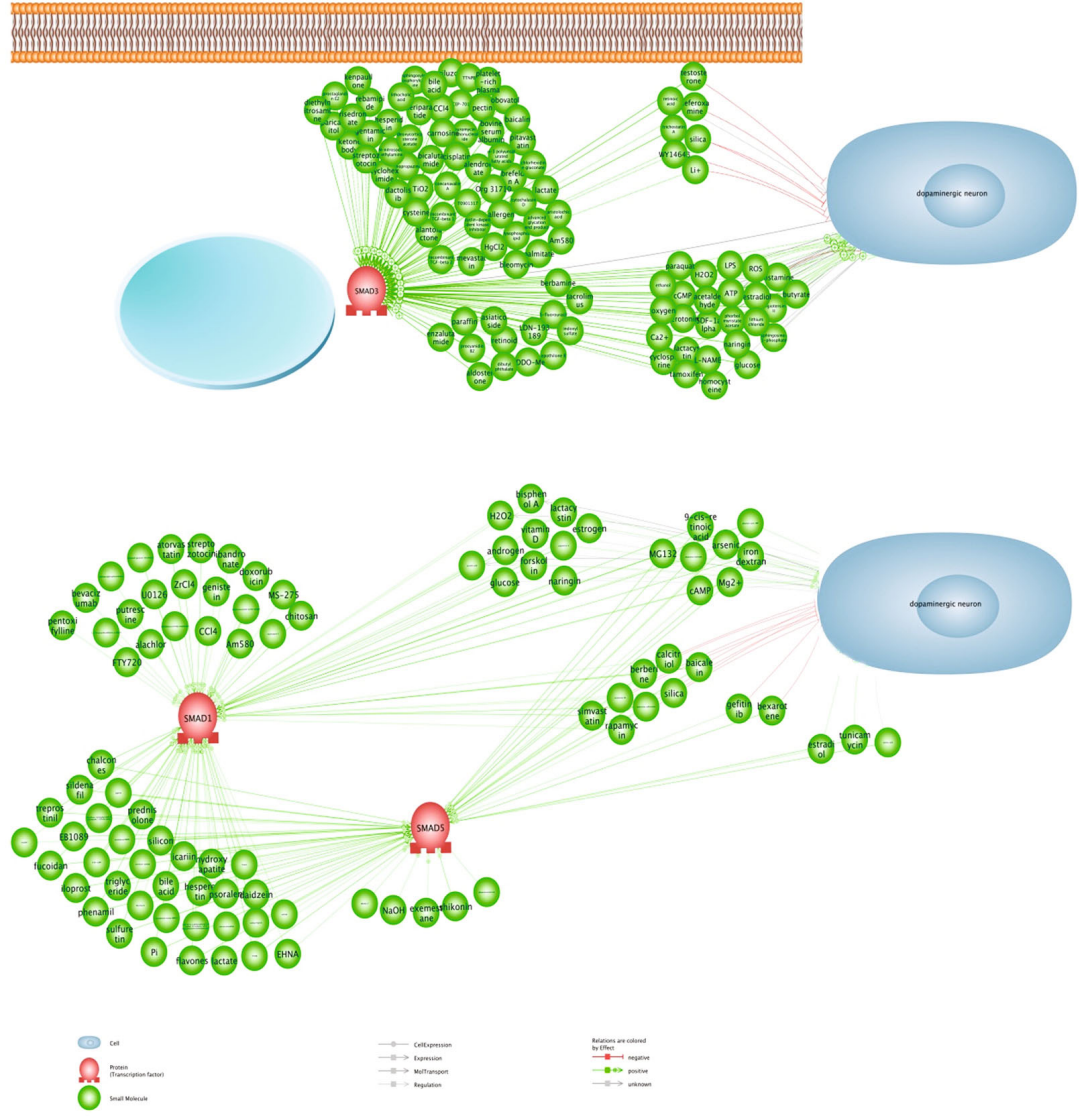
Common small molecules between neurogenesis and osteogenesis that function by focusing on the Smad signaling pathway. Drawn by Pathway Studio.

Notably, all effects may be considered dose-dependent and act differently in the two systems at different concentrations. It is worth noting that although R-Smad activation and Smurf phosphorylation are helpful in Parkinson's disease therapeutic procedures, uncontrolled overexpression of R-Smads and co-Smad in cells might result in tissue fibrosis, and their dysfunctionality leads to cancer (Figure 4). Therefore, dose-controlled small-molecule therapy is important in Parkinson's disease therapeutic studies involving osteogenic small molecules.

## Figures and Tables

**Table 1:** Crosstalk of osteogenic small molecules involve in Smad1/3/5, BMP2, and Smurf1 signaling pathway.

	Small molecule	Parkinson's disease
Smad3 and p-Smad3	Pitavastatin	May apply
	Mevastatin	May apply
	Simvastatin	May apply
	Sirolimus	May apply

Smad1/5 and p-Smad1/5	Isoliquiritigenin	May apply
	4′-hydroxychalcone	May apply
	FK506	May apply
	PGE-5516909	May apply

Smurf1 inhibitors	C22H20ClF3N4O3S	Not apply
	C25H26FN3O4	Not apply
	Phenamil	Not apply

BMP2	2-((1-(benzyl(2-hydroxy-2-phenylethyl)amino)-1-oxo-3-phenylpropan-2-yl)carbamoyl) benzoic acid	May apply
